# Evaluation of Antioxidant and Antiproliferative Properties of *Cornus mas* L. Fruit Juice

**DOI:** 10.3390/antiox8090377

**Published:** 2019-09-05

**Authors:** Angeliki Tiptiri-Kourpeti, Eleni Fitsiou, Katerina Spyridopoulou, Stavros Vasileiadis, Christos Iliopoulos, Alex Galanis, Stavroula Vekiari, Aglaia Pappa, Katerina Chlichlia

**Affiliations:** 1 Department of Molecular Biology and Genetics, Democritus University of Thrace, University Campus, Dragana, 68100 Alexandroupolis, Greece; 2 ELGO-DEMETER, Institute of Technology of Agricultural Products, 14123 Lykovrisi, Athens, Greece

**Keywords:** cornelian cherry, chemical composition, antioxidant, anticancer, antitumor, oral administration

## Abstract

*Cornus mas* L. (Cornelian cherry) is a flowering plant indigenous to Europe and parts of Asia, mostly studied for the antimicrobial activity of its juice. In this report, we investigated the composition and the in vitro antioxidant capacity of *Cornus mas* L. fruit juice from Greece, as well as its antiproliferative properties in vitro and in vivo. The fruits showed a high content of citric, malic, and succinic acid, in contrast to their juice, which had a low concentration of organic acids. The juice demonstrated significant antioxidant activity against the free radical 2,2-diphenyl-1-picrylhydrazyl (DPPH) and modest antiproliferative potential against four human cancer cells lines and one murine: mammary adenocarcinoma MCF-7, hepatocellular carcinoma HepG2 and colon adenocarcinomas Caco2, HT-29, as well as murine colon carcinoma CT26. Cell viability was reduced by 40–50% following incubation of the cells with the highest concentration of the juice. Although Cornelian cherry juice exhibited in vitro growth inhibitory effects against colon carcinoma cells, no tumor growth inhibition was observed in an in vivo experimental colon carcinoma model in mice following prophylactic oral administration of a daily dose of 100 μL juice for a period of 10 days. Thus, our findings raise interesting questions for further research on *Cornus mas* L. fruit juice, and in parallel, the strong antioxidant potential implies that the plant could be further explored and exploited for its protective effect against oxidative damage.

## 1. Introduction

*Cornus mas* L. (European Cornelian cherry) is a flowering plant that is indigenous to southern and central Europe and southwest Asia and belongs to the Cornaceae family [1]. Most of the species of this family are used for decorative purposes and only some produce fruit, including *Cornus mas* [2]. It is a tall deleterious shrub or small tree with dark brown branches and greenish twigs [3]. The fruit is an oblong, red drupe, containing a single seed. It is used for food, syrup and jam production and, lately, also fermented for the production of a low alcoholic functional beverage with high nutritional value [4,5]. Traditionally, it has been mainly used to comfort gastrointestinal health conditions [6]. In Greece, in particular, it has also been used for skin diseases, tuberculosis and menstrual problems [7]. In Europe, fruit extracts have been used for cosmetic purposes [8].

Nowadays, the plant, mainly the fruit and leaves, in the form of dried powder or methanolic extracts, has been studied for a variety of biological properties, exhibiting promising activity against, for example, diabetes, different microbial strains, inflammation and oxidative stress (reviewed in [7]). There are also data on the fruit’s role against cancer [2,9], findings that are regarded as very important considering the need for alternative nontoxic treatments to the commercially available chemotherapeutic drugs that exhibit significant toxicity.

Cornelian cherry fruits are a good source of bioactive natural health-promoting compounds. As previously reported, fruits are rich in polyphenols (anthocyanins, cinnamic acids, flavonoids, benzoic acids, catechins and tannins) showing a prevalence (37.36%), followed by monoterpenes and organic acids in similar proportions (26.3% and 25.9%, respectively) and vitamin C (10.7%) [10]. However, as known, the content of phytochemicals has considerable compositional variations in the same species, according to the genotypes and experimental factors, such as type of cultivation, year and location of the sampling and meteorological conditions.

In the present study, the juice of fruits from *Cornus mas* L. from Greece was examined for its composition. We also investigated the in vitro antioxidant and antiproliferative properties of the juice and its antitumor capacity in vivo. To our knowledge, this is the first report investigating the in vitro and in vivo effect of *Cornus mas* juice in an in vivo experimental model of colon carcinoma.

## 2. Materials and Methods

### 2.1. Compositional Analysis of Cornelian Cherry (Cornus mas)

In the present study *Cornus mas* L. fruits of “Skinitsa” variety, cultivated in Veria area, Greece, were used. The *Cornus mas* L. fruits of full maturity were obtained from a commercial field and the year of sampling was the year when the project started (2012). Colour parameters were determined in healthy fruits, mineral nutrient, sugar and organic acid contents were measured in the flesh and juice, and the fatty acid composition in the kernel.

The Cornelian cherry fruit skin colour from healthy fruits was measured by the use of a portable Minolta colorimeter. Colour parameters L*, a*, and b* were determined by the CIELAB colour space, defined by the International Commission on Illumination (CIE), where L* indicates lightness and a* and b* are chromaticity coordinates; a* and b* are colour directions: +a* is the red axis, −a* is the green axis, +b* is the yellow axis and −b* is the blue axis. Water content was determined according to the official method of AOAC (Association of Official Agricultural Chemists) International [11].

Preparation of fruits and fruit juice: Fruits collected in full maturity were washed, cut, the kernel was removed and the rest of the material, the pulp, was lyophilized and kept under −4 °C until use. In this lyophilized material, the mineral nutrient, sugar and organic acid contents were measured. The fruit juice was extracted from a different batch of fresh fruits. Cornelian cherry fruits were pressed through a laboratory press to obtain the fruit juice. Juice was kept frozen until it was further analysed for acidity, pH and Brix, as well as later for examining the antioxidant and antiproliferative activity.

Fat composition: Lyophilized fruits were used for fat extraction by the Soxhlet method.

Fatty acids analysis: The fatty acid analysis was done on dried fruit kernels. The fruit kernels were removed from the fruits, were grounded and their oil was extracted by the Soxhlet apparatus through the method of AOAC (991.31.1997) [11]. The fatty acid composition and content (%) were analysed by gas chromatography-mass spectrometry (GC-MS) (Model Varian CP-3800, equipped with flame ionization detector, Thermo Fisher Scientific, Waltham, MA, USA), after methylation with boron trifluoride for fatty acid methyl esters (FAMEs) as described previously [12]. The FAMEs were suspended in hexane and analysed by gas chromatography with an Agilent DB23 capillary column (Model No. 123-2332, 30.0 m × 0.32 mm × 0.25 μm, Agilent J and W Scientific, Santa Clara, CA, USA). Helium gas was used as a carrier gas with a column flow rate of 2.0 mL/min.

For the analysis, the following set-up conditions were used: Initial oven temperature was set at 150 °C, held for 18 min, subsequently rammed to 185 °C at a rate of 5 °C/min. Then the oven temperature was increased to 210 °C at a flow rate of 5 °C/min and held for 2 min, and then further increased to 240 °C at a rate of 10 °C/min. The injector and flame ionization detector temperatures were set at 260 and 270 °C, respectively. Individual fatty acids were identified by comparison of their retention times with external standard (Supelco 37 Component FAME Mix, CRM47885, Sigma-Aldrich, St. Louis, MO, USA) retention times. The amounts of individual fatty acid methyl esters identified were obtained from the chromatogram areas and expressed in percentage of the total fatty acids.

Organic acid analysis: Sugar and organic acid analyses were performed in the received juice. Organic acid analysis was also performed in the lyophilized fruits. Sugars and organic acids were analysed simultaneously by high performance liquid chromatography (HPLC model Agilent 1100, equipped with UV-MWD and RI detector, Agilent, Santa Clara, CA, USA). The separation was carried out on Aminex HPX-87H cation-exchange columns (300 × 7.8 mm I.D., Bio-Rad Laboratories, Hercules, CA, USA). The eluent was 4.5 mM sulphuric acid. Flow-rate was 0.7 mL/min, the column temperature was set at 65 °C and injection volume was 20 μL. The samples, after they were pitted, were lyophilized. Then they were diluted with water, centrifuged, filtered through 0.45 μm and injected into the HPLC system. The sugars and organic acids were determined using the external standard method.

### 2.2. Cell Lines and Cell Cultures

The human hepatocellular carcinoma HepG2, the human breast adenocarcinoma MCF-7, the human colon adenocarcinomas Caco2 and HT-29 were obtained from the American Type Culture Collection (ATCC) (Rockville, MD, USA). Murine colon carcinoma CT-26 was kindly provided by V. Schirrmacher (DKFZ, Heidelberg). HepG2, MCF-7, HT-29 and CT-26 cells were grown and maintained in Dulbecco’s modified Eagle’s medium (DMEM) (Gibco, Thermo Fisher Scientific, Waltham, MA, USA), while Caco2 cells were grown and maintained in RPMI-1640 medium (Gibco, Thermo Fisher Scientific, Waltham, MA, USA), both supplemented with 10% fetal bovine serum (FBS) (Biosera, Boussens, France), penicillin (100 U/mL), and streptomycin (100 mg/mL) (Biosera, Boussens, France) and were incubated at 37 °C in a humidified atmosphere of 95% O_2_ and 5% CO_2_. Stock cultures were passaged at 2 to 3-day intervals. For the Sulforhodamine B (SRB) assay, cells were seeded at a density of 5 × 10^3^ cells/well in 96-well plates.

### 2.3. Determination of Cornus mas L. Juice in Vitro Antioxidant Activity (DPPH Assay)

The radical scavenging activity of Cornelian cherry juice was estimated using the free radical 2,2-diphenyl-1-picrylhydrazyl (DPPH) assay, as previously described with few modifications [13]. Different concentrations of the juice (0.02–0.2% *v*/*v*) were prepared using water as the solvent. Ten microliters of each concentration were placed in a 96-well plate and 190 μL of 300 μM methanolic solution of DPPH (Calbiochem, Darmstadt, Germany) were added. Ten microliters of water (dH_2_O) with 190 μL DPPH were used as the control. The plate was left in darkness for 30 min and then the absorbance was measured at 517 nm using an absorbance microplate reader (Sunrise, Tecan, Männedorf, Switzerland). All determinations were performed in triplicates.

The percentage inhibition of the DPPH radical for each concentration was determined by making use of the following formula: % DPPH radical scavenging activity = [(OD_control_ − OD_sample_)/OD_control_)] × 100.

### 2.4. Determination of the In Vitro Antiproliferative Effect of Cornus mas L. Juice (Sulforhodamine B Assay)

The viability of cancer cells after treatment with the juice was determined using the Sulforhodamine B (SRB) assay, as previously described with few modifications [14]. SRB is a dye that binds to basic amino acids of cellular proteins and, then, the number of viable cells is estimated with colorimetric evaluation [15]. Cells were plated in 96-well plates and treated with different concentrations of the juice (0.0007–1% *v*/*v*). Then, the cells were fixed with the addition of 25 μL of 50% (*w/v*) cold trichloroacetic acid (TCA) (Applichem, Darmstadt, Germany) to the growth medium and incubation of the plates at 4 °C for 1 h. The cells were washed five times with tap water and then stained with 50 μL of 0.4% (*w/v*) SRB dye (Sigma-Aldrich, St Louis, MO, USA) in 1% (*v*/*v*) acetic acid (Scharlau, Barcelona, Spain) for 30 min at room temperature. Next, the cells were rinsed five times with 1% (*v*/*v*) acetic acid to remove the unbound dye. The fixed, stained plates were allowed to air-dry and afterwards the bound dye was solubilized by adding 100 μL of 10 mM Trizma base (Sigma-Aldrich, St Louis, MO, USA) for at least 5 minutes. Absorbance was measured at 570 nm using an ELISA plate reader (Sunrise, Tecan, Männedorf, Switzerland) and the percent cellular survival was calculated using the formula:

[(Sample OD_570_ − media blank OD_570_)/(mean control OD_570_ − media blank OD_570_)] × 100 (1)

### 2.5. Animals and CT26 Experimental Tumor Model

In order to examine the in vivo antitumor effect of the *Cornus mas* L. juice against colon cancer, an experimental BALB/c colon cancer model was used as previously described [16]. Female BALB/c mice (6–8 weeks old, weight 20–25 g) were purchased from the Animal Facility of Pasteur Institute (Athens, Greece) and kept in the Animal House of Medical School at the University of Ioannina (Greece). Mice were housed in polycarbonate cages, max. 10 mice per cage, at room temperature, on a 12 h light–12 h dark cycle and were provided with tap water ad libitum and a commercial pelleted diet (Mucedola). The experimental protocol was approved by the Animal Care and Use Committee of the Veterinary Service in Ioannina and was in compliance with Directive 86/609/EEC. Female BALB/c mice were separated into two independent groups (10 mice per group) and received daily 100 μL *C. mas* juice with oral gavage for a period of 10 days. At day 10, 5 × 10^6^ CT26 colon cancer cells were administered subcutaneously as a single dose and 8 days later mice were euthanized by cervical dislocation and tumors were harvested. Tumor dimensions were determined by an electronic micro-meter and tumor volume (mm^3^) was calculated by the modified ellipsoid formula: 0.5 × (width^2^ × length). In the control group, BALB/c mice received an equal volume of phosphate buffered saline (PBS). Animals were weighted daily and monitored for signs of disease or discomfort.

### 2.6. Statistical Evaluation

All data were analysed with Sigmaplot v.10 software (Systat software GmbH, Erkrath, Germany).

The IC_50_ and EC_50_ values (Inhibitory Concentration that causes a 50% reduction in the DPPH free radical; Efficient Concentration that causes a 50% decrease in cancer cells viability) were calculated from the respective dose response curves by regression analysis using a four-parameter logistic curve.

For in vivo experiments, statistical difference was evaluated by performing independent samples Student’s *t*-test (normal distribution). Differences between control and treated group were considered significant when *p* < 0.05.

### 2.7. Ethics Statement

Animal experiments were approved by the Animal Care and Use Committee of the Veterinary Department of Ioannina Prefecture (license number EL20BIO02) since it complied with the requirements set by Directive 86/609/EEC and PD 160/91, which was the legislation in force at the time of experimentation. All animal experiments were conducted in light of the three R’s (replacement, refinement, reduction) and all mice used for the experiments were not subjected to pain or discomfort.

## 3. Results and Discussion

### 3.1. Composition of Cornus mas L. Juice

Content of organic acids (mg/g) in *Cornus mas* juice and in the lyophilized fruit was determined. Juice was prepared from a number of fruits, while from other fruits, after the removal of the kernel, the pulp was lyophilized for analysis. *Cornus mas* presented high content of organic acids while a small amount was transported in its juice. The organic acids with the largest proportion in its flesh were citric, malic, and succinic, malic acid presenting the largest proportion, while the same acids per gram were detected in the juice but in less quantity (Table 1). These results are in agreement with previous studies showing a prevalence for malic acid in juice from fruits picked from trees in southern Poland; however, the Greek fruit juice showed almost 50% less malic acid than the Polish juice [17].

Colour parameters were measured on the skin of whole and healthy fruits. For the brightness, the L* value, which represents the lightness coordinate, was 27.1. A positive a* value, that represents intensity of red colour, was 23.3, a value fairly high. The value b* was only 8.1 (Table 2). Consumer acceptability of Cornelian cherry fruit depends on a number of parameters, although skin colour is often regarded as the one according to which the consumers make the first choice of whether to buy them or not. Colour characteristics like brightness, redness, yellowness, and shine are among characteristics that can trigger consumer willingness at first glance. Interestingly, the percentage of fat in fruits was 0.25%.

The weight of 50 Cornelian cherries was 133.66 g, while the mean weight of flesh was 2.67 g. Brix, pH and acidity were also measured for the juice and the results are presented in Table 3. Mineral composition of Cornelian cherry flesh is presented in Table 4. As shown, the most abundant mineral present was potassium followed by calcium and magnesium. The main sugars were found to be glucose and fructose.

Fatty acid composition of kernels is presented in Table 5. Six fatty acids were identified in kernels. The most abundant fatty acids were unsaturated linoleic acid and oleic acid, followed by linolenic acid. Among the saturated fatty acids, the prevailing acid was palmitic acid, followed by stearic and arachidonic. The main fatty acid was linoleic, which represented 60% of all the fatty acids. Oleic acid represented 30%, while palmitic was 8%. The data from our investigation are in good agreement with those reported by Vidrih et al. [18].

### 3.2. Antioxidant Activity of Cornus mas L. Juice In Vitro

**Figure 1:**
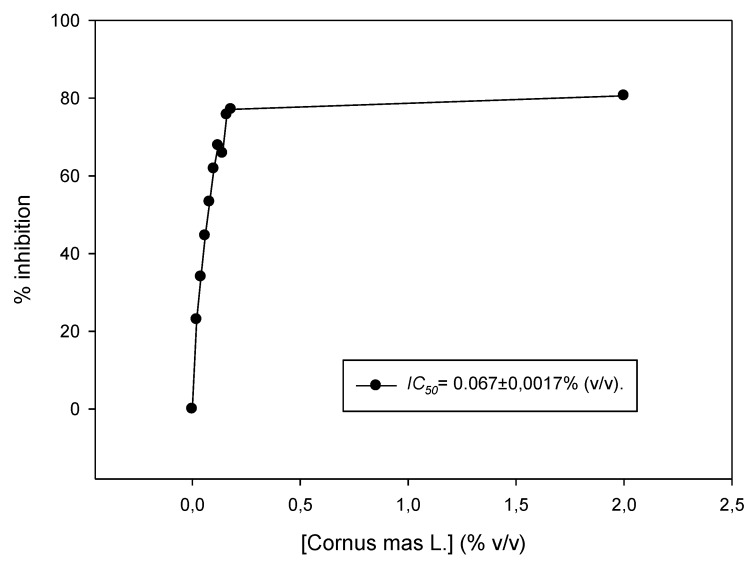
DPPH (2,2-Diphenyl-1-picrylhydrazyl) radical scavenging activity of *Cornus mas* L. juice. Increasing concentrations of the juice (0.02–0.2% *v*/*v*) were incubated in the presence of DPPH for 30 min. Representative figure of at least three independent experiments.

The juice was tested for its antioxidant capacity in vitro using the DPPH assay. The IC_50_ value was estimated to be 0.067% ± 0.001% (*v*/*v*) (Figure 1). In another study, Cornelian cherry juice showed significant antioxidant activity in vitro using the FRAP (Ferric Reducing Antioxidant power) method (23.5 mmol FeL) compared to other well-known fruit juices, and correlated with its content in polyphenols and other biological compounds [17]. Moreover, liquors made from Cornelian cherries were characterized by high total acidity and antioxidant capacity (5064.2 μΜ Trolox/100 mL using the ABTS (2,2’-Azino-bis(3-Ethylbenzothiazoline-6-Sulfonic acid) enzymatic assay) [18]. It was also found that Cornelian cherries from Greek cultivars had the highest content in vitamins C and E compared to other Greek cultivars [19,20], that, together with other components rich in *C. mas* cherries (e.g., anthocyanins including delphinidin 3-*O*-beta-galactopyranoside, cyanidin 3-*O*-beta-galactopyranoside and pelargonidin 3-*O*-beta-galactopyranoside), confer the potent activity of the fruits [8,10,21]. It is important to note that there are many methods to assess the antioxidant capacity of an agent, and results are difficult to compare, since different protocols and units are used. Interestingly, the antioxidant activity and the bioaccessibility of anthocyanins of a Cornelian cherry fruit extract were also evaluated in an in vitro human gastrointestinal digestion model [22].

### 3.3. Antiproliferative Effect of Cornus mas L. Juice In Vitro against Cancer Cell Lines

**Figure 2:**
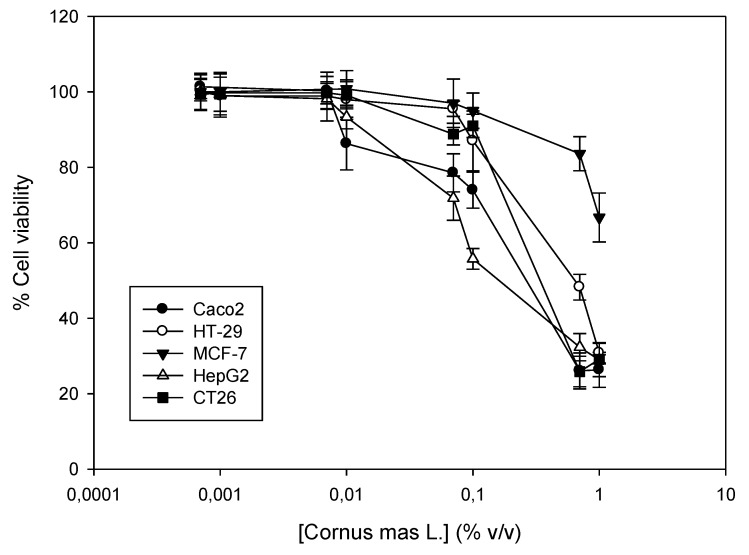
Antiproliferative activity of *Cornus mas* L. juice against a panel of four human cancer cell lines and one from mice. Human Caco2, HT-29, MCF-7 and HepG2 and murine CT26 cells were incubated with increasing concentrations of the juice (0.0007–1% *v*/*v*) for 72 h. A dose-dependent estimation of cell viability was determined by the Sulforhodamine B (SRB) assay. Representative figure of at least three independent experiments.

Cornelian cherry juice was tested for its cytotoxic activity against a panel of five cancer cell lines. Cells were incubated with increasing concentrations of the juice for 72 h (0.007–1% *v*/*v*) and then, cell viability was determined using the SRB assay. To our knowledge, this is the first time that the in vitro antiproliferative properties of the juice are described, although there are data on the cytotoxic activity of alcoholic and methanol extracts against cancer cells [9,23]. Our results indicate that the juice is most potent against the human hepatocellular carcinoma HepG2, followed by the murine colon carcinoma CT26. The human colon carcinomas Caco2 and HT-29 exhibited similar sensitivity, which was weaker than their murine counterpart and was approximately four- to six-fold weaker in comparison to HepG2 cells. Finally, the breast adenocarcinoma MCF-7 exhibited the highest resistance to the action of the juice, where cell viability did not decrease more than 50% compared to the control samples (Figure 2, Table 6).

Previous studies have been conducted on the in vitro cytotoxic effect of a hydro-alcoholic extract of *Cornus mas* fruit, which showed that the extract possesses high potency to inhibit proliferation of different tumor cells (A549, lung non-small cell cancer; MCF-7, breast adenocarcinoma; SKOV3, ovarian cancer; PC-3, prostate adenocarcinoma) in a dose-independent manner, suggesting that an optimal biological dose is more important and relevant than a maximally tolerated one [9]. Additionally, preliminary results showed that methanol extracts of leaves and flowers of *Cornus mas* possess potential cytotoxic activity against human cancer cell lines in vitro, exhibiting a more potent effect against HeLa cells compared to LS174 cells [23]. These results indicate that the differences in the *Cornus mas* alcoholic and methanol extracts’ activity, as well as our results on the juice’s activity, may be due to potential differences in phenolic and organic acid contents. Further work is needed to clarify the role of the constituents.

Teller et al. showed that the anthocyanidin delphinidin, which is present in *Cornus mas* L., inhibits activation of ErbB and vascular endothelial growth factor receptor family members [24]. Recently, it was demonstrated that delphinidin blocks the proliferation of primary human blood and lymphatic endothelial cells as well as carcinoma cells in vitro in a dose-dependent manner. At higher concentrations, apoptosis of endothelial and tumor cells was induced [25].

### 3.4. Effect of Cornus mas L. Juice on the Growth of Syngeneic Colon Carcinoma Tumors 

The in vivo antitumor effect of the *Cornus mas* L. juice was examined against an experimental colon cancer model in BALB/c mice. This model was chosen as a more relevant model for dietary factors, because many natural compounds, phenolics, flavonoids and particularly their subclasses, anthocyanins, are highlighted to play an important role in human medical nutrition. Flavonoids are a key regulator between diet and chronic diseases. Microbial enzymes cleave flavonoids in the intestine which further metabolize in colon and liver. Thus, colorectal cancer appears most relevant to dietary factors [26].

**Figure 3:**
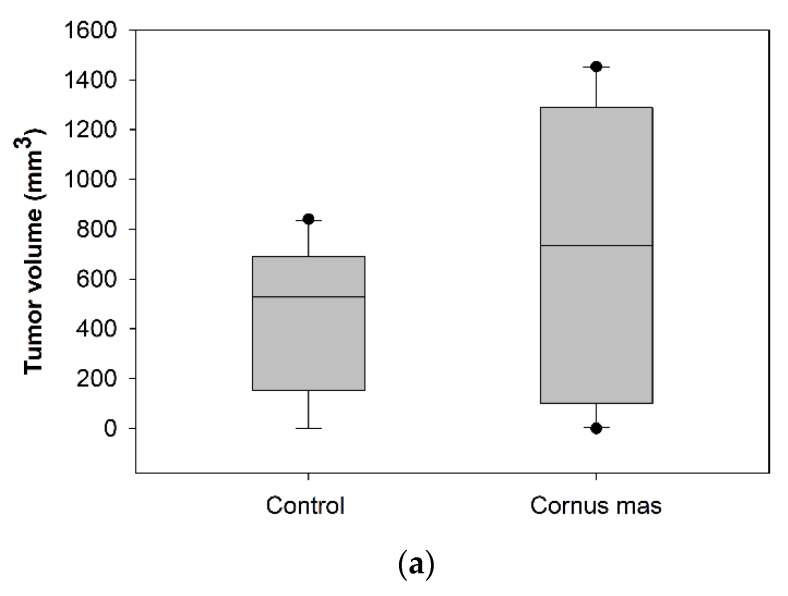

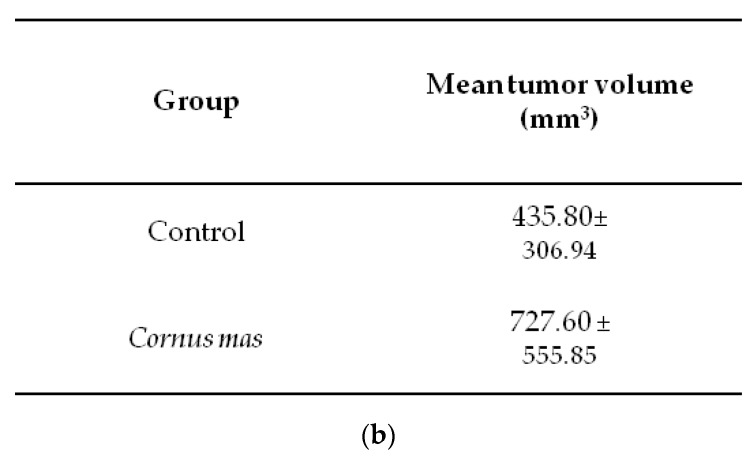
Effect of *Cornus mas* L. juice to CT26 colon carcinoma. BALB/c mice received *Cornus mas* L. juice for 10 days per os with oral gavage and were injected subcutaneously with 5 × 10^6^ CT26 colon cancer cells. Eight days later, BALB/c mice were euthanized and tumors were harvested; (**a**) table shows tumor volume (mm^3^) and incidence (%) in BALB/c mice receiving *C. mas* L. juice (treated group) or phosphate buffered saline (PBS) (control group). Mean tumor volume did not differ significantly between the groups under study (*p* = 0.164); (**b**) box-plots display the distribution of tumor volume (mm^3^) in the control and treated group of BALB/c mice.

Female BALB/c mice received daily 100 μL *C. mas* juice (treated group) or PBS (control group) per os with oral gavage for 10 days. At day ten, 5 × 10^6^ CT26 colon cancer cells were administered subcutaneously and eight days later mice were euthanized to define tumor incidence and growth. No significant statistical difference was observed in tumor volume (*p* > 0.05) or tumor incidence between the control and the Cornelian cherry juice groups (Figure 3a). As shown in the box-plot in Figure 3b, tumor volumes of mice in the control and juice group display similar (or even higher) tumor volume distribution. We conclude that under the examined experimental conditions short-term oral administration of Cornelian cherry juice provides no prophylactic effect in mice bearing colon cancer.

Thus, although our results show a *Cornus mas*-induced antiproliferative effect in vitro against CT26 colon cancer cells, this is not the case in vivo. Under the above-mentioned experimental settings there was no tumor growth inhibition of CT26 tumors in mice following oral administration of *Cornus mas L.* juice. This could be due to a low dosage of juice (100 μL) used or a short time (10 days) of juice administration, low bioavailability or potential different molecular mechanisms that are induced in vivo following juice administrations, as related to the in vitro situation in cultured cells. Noteworthy, Moldovan et al. showed that a Cornelian cherry fruit extract induced anti-inflammatory activity using an in vivo model of paw inflammation in Wistar rats [21]. Interestingly, in another study in vivo administration of delphinidin significantly reduced angiogenesis in the MT-450 rat syngeneic breast tumor model, although tumor growth and metastasis were promoted. The antiproliferative effect of delphinidin on cultured cells does not necessarily reflect the response of tumors to this anthocyanidin in vivo [25].

## 4. Conclusions

Cornelian cherry (*Cornus mas* L.) juice was investigated for its composition and also for its antioxidant and antiproliferative properties. The fruits presented high content of citric, malic, and succinic acids, in contrast to their juice, which had a low concentration of organic acids. Total lipid content was low, while the main sugars found were glucose and fructose. In kernels, the most abundant fatty acid was unsaturated linoleic acid followed by oleic acid, while the most abundant mineral in lyophilized *C. mas* L. was potassium. The juice demonstrated significant antioxidant but moderate antiproliferative capacity against a panel of human and murine cancer cell lines. Although Cornelian cherry juice induced an antiproliferative effect in vitro against colon carcinoma cells, no tumor growth inhibition was observed in an in vivo experimental mouse tumor model of colon carcinoma following short-term oral administration of the juice. These results raise interesting questions for further research, and in parallel indicate that the plant could be further explored and exploited for its protective effect against oxidative damage.

## Figures and Tables

**Table 1:** Content of organic acids (mg/g) in *Cornus mas* juice and in the lyophilized *Cornus mas* fruit.

Sample	Citric Acid	Malic Acid	Succinic Acid
Fruit	0.2	74.4	21.8
Juice	0.1	14.5	6.8

**Table 2:** Colour parameters, moisture and fat content (%) in *Cornus mas* L. fruits.

Colour * (CIELAB Parameters)			Dry Material	Moisture	Fat
L*	a*	b*	%	%	%
27.1	23.3	8.1	24.75	75.25	0.25

* The colour was measured on the skin of whole and healthy fruits by the CIELAB colour space. L*, CIE lightness coordinate; a*, CIE red(+)/green(−) colour attribute; b*, yellow(+)/blue(−) colour attribute.

**Table 3:** Analysis of *Cornus mas* juice.

Brix	pH	Acidity (%) *
21.40	3.27	2.59

* The acidity is expressed as citric acid equivalents.

**Table 4:** Content of minerals (mg/100 g) or sugars (%) in lyophilized *Cornus mas*.

Minerals					Sugars (%)	
Κ	Fe	Cu	Ca	Μg	Fructose	Glucose
672.44	0.58	0.38	173.41	52.02	20.3	31.2

**Table 5:** Composition of *Cornus mas* kernel oil in fatty acids (%).

Fatty Acids	Percentage of Fatty Acids in *Cornus mas* Kernel Oil
Palmitic	7.0
Stearic	2.2
Oleic	23.0
Linoleic (ω-6)	53.6
α-linolenic (ω-3)	1.4
Arachidonic	1.4

**Table 6:** Half maximal Effective Concentration EC_50_ (%*v*/*v*) values for Cornelian cherry juice against the human cancer cell lines Caco2, HT-29, MCF-7 and HepG2 as determined by the SRB assay.

Cell Lines	EC_50_ (% *v*/*v*)
Caco2	0.37 ± 0.04
HT-29	0.49 ± 0.09
MCF-7	n.d.*
HepG2	0.08 ± 0.004
CT26	0.20 ± 0.08

* *n.d*.: not determined.
